# Intra-Specific Latitudinal Clines in Leaf Carbon, Nitrogen, and Phosphorus and their Underlying Abiotic Correlates in *Ruellia Nudiflora*

**DOI:** 10.1038/s41598-017-18875-w

**Published:** 2018-01-12

**Authors:** Luis Abdala-Roberts, Felisa Covelo, Víctor Parra-Tabla, Jorge C. Berny Mier y Terán, Kailen A. Mooney, Xoaquín Moreira

**Affiliations:** 1 Departamento de Ecología Tropical, Campus de Ciencias Biológicas y Agropecuarias, Universidad Autónoma de Yucatán, Apartado Postal 4-116 Itzimná, 97000 Mérida, Yucatán, Mexico; 2 Departamento de Sistemas Físicos, Químicos y Naturales, Universidad Pablo de Olavide, Carretera de Utrera km. 1, 41013 Sevilla, Spain; 3 Department of Plant Sciences, University of California-Davis, One Shields Avenue, Davis, California, 95616 USA; 4 Department of Ecology and Evolutionary Biology, University of California, Irvine, California, 92697 USA; 5 Misión Biológica de Galicia (MBG-CSIC), Apdo. 28, 36080 Pontevedra, Spain

## Abstract

While plant intra-specific variation in the stoichiometry of nutrients and carbon is well documented, clines for such traits have been less studied, despite their potential to reveal the mechanisms underlying such variation. Here we analyze latitudinal variation in the concentration of leaf nitrogen (N), phosphorus (P), carbon (C) and their ratios across 30 populations of the perennial herb *Ruellia nudiflora*. In addition, we further determined whether climatic and soil variables underlie any such latitudinal clines in leaf traits. The sampled transect spanned 5° latitude (ca. 900 km) and exhibited a four-fold precipitation gradient and 2 °C variation in mean annual temperature. We found that leaf P concentration increased with precipitation towards lower latitudes, whereas N and C did not exhibit latitudinal clines. In addition, N:P and C:P decreased towards lower latitudes and latitudinal variation in the former was weakly associated with soil conditions (clay content and cation exchange capacity); C:N did not exhibit a latitudinal gradient. Overall, these results emphasize the importance of addressing and disentangling the simultaneous effects of abiotic factors associated with intra-specific clines in plant stoichiometric traits, and highlight the previously underappreciated influence of abiotic factors on plant nutrients operating under sharp abiotic gradients over smaller spatial scales.

## Introduction

The abundance of key elements such as nitrogen (N), phosphorus (P) and carbon (C) in plant tissues exerts strong controls over plant physiological rates and ecological processes such as primary productivity, nutrient decomposition and cycling, and herbivory^1–5^. At the same time, multiple abiotic factors (e.g., temperature, precipitation, soil weathering) influence the concentration of these elements in plant tissues^6^. Therefore, understanding how and which of these abiotic factors influence element abundances is key for predicting the effects of global change on ecosystem function^7,8^.

The concentrations of C, N, P and their relative amounts (N:P, C:N, C:P) in plants are influenced by factors such as plant growth rate and biomass, soil biogeochemistry, and plant species composition^6,7,9^. For instance, higher plant growth rates are associated with increased allocation to P-rich RNA and enzymes involved in P metabolism to support increased growth rates^7,9^. In addition, plant N and P are influenced by soil nutrient availability, where for example N is thought to be more limiting for plants in younger soils, whereas P tends to be more limiting on older soils which are typically P-poor due to physical processes such as weathering and leaching^6,9^. Within this context, the study of latitudinal gradients has provided a useful framework for understanding the drivers of plant N, P and C stoichiometry^7^, as well as functional traits closely linked with nutrient use and allocation^10,11^. This is because many of the factors that influence plant nutrients such as soil age and weathering (greater in tropical vs. temperate zones), plant growth rates (greater in the tropics), and climatic factors (directly or indirectly) cause shifts in plant nutrient concentrations and their relative abundances. Global analyses across hundreds of plant species have shown that plant N generally increases (or remains unaltered) whereas plant P decreases towards lower latitudes^6,7^. As a result of these individual patterns for each element, N:P ratios usually increase with temperature towards lower latitudes^6,7,12^. Research thus far has contributed to a better understanding of abiotic controls on latitudinal variation in plant nutrients and stoichiometry, which may in turn provide insight into the drivers of ecosystem function and plant community responses to abiotic gradients.

Although much of the work on gradients in plant stoichiometry has been conducted at broad spatial scales and across many plant species (reviewed by Elser *et al*.^1^ and Enquist *et al*.^7^), another line of studies has focused on ecological gradients in plant intra-specific variation in functional traits^13–15^ and, to some extent, plant stoichiometry^16–18^. Intra-specific latitudinal gradients in plant nutrients are strongly shaped by phenotypic plasticity in response to environmental variation, but may also be caused by genetic variation in nutrient allocation and plant physiology due to plant adaptation to the environment^7^. Research has shown that although intra-specific comparisons necessarily involve smaller latitudinal ranges, species’ distributions may still span and adapt to these ecological clines^13,19,20^. For example, studies on intra-specific variation along ecological gradients have provided direct evaluations of how plant traits evolve along biotic and abiotic gradients^13,16,19^. A better understanding of intra-specific clines in plant nutrient stoichiometry may help link evolutionary change with ecosystem function to the extent that the evolution of plant traits alters nutrient requirements and this in turn influences biogeochemical processes^21^. In turn, abiotic factors may influence evolutionary change by exerting controls over nutrients used in the biochemical machinery^21,22^. The study of intra-specific variation in plant stoichiometric traits therefore offers a bridge for understanding the relationships between evolutionary change and ecosystem function.

We tested for latitudinal variation in leaf C, N, and P concentrations and their ratios across 30 populations of the perennial herb *Ruellia nudiflora* (Engelm. and Gray) Urb. (Acanthaceae), and further examined whether climatic and soil variables were associated with any such latitudinal clines. The sampled transect spanned 5° latitude (ca. 900 km) from northern Yucatan (Mexico) to southern Belize and covered one-third of the species’ latitudinal range. In addition, it exhibited a four-fold precipitation gradient and 2 °C variation in mean temperature which represented the entirety and one-third of the precipitation and temperature gradient (respectively) within this species’ distribution range^23^. We previously reported on patterns of herbivory and defences (trichomes, phenolic compounds) for *R*. *nudiflora* along this transect using the same populations^20,23^. Here we now ask whether (*i*) there is a latitudinal gradient in leaf C, N, P, and their ratios, and (*ii*) whether abiotic factors, namely climatic variables and soil conditions, are associated with geographic variation in leaf nutrients and potentially explain any such latitudinal clines in these plant traits. In addressing the above, the present work delivers a unique evaluation of the independent and combined influences of abiotic factors underlying intra-specific clines in plant nutrients and stoichiometry.

## Results

### Population variation in leaf nutrients

Results from the GLM analyses indicated substantial variation among populations in the studied leaf traits. Specifically, we found significant population variation in the concentration of leaf P (F_29,90_ = 6.64, *P* < 0.0001; range: 0.77 ± 0.37 mg g^−1^ to 2.59 ± 0.42 mg g^−1^), N (F_29,90_ = 6.34, *P* < 0.0001; range: 21.29 ± 1.87 mg g^−1^ to 37.97 ± 3.21 mg g^−1^), and C (F_29,80_ = 2.51, *P* = 0.0007; range: 282.70 ± 4.59 mg g^−1^ to 338.79 ± 9.17 mg g^−1^). In addition, results for element ratios indicated significant population variation in C:N (F_29,80_ = 3.63, P < 0.0001; range: 8.01 ± 1.28 to 14.41 ± 0.91), in C:P (F_29,80_ = 12.40, P < 0.0001; range: 135.07 ± 24.34 to 428.39 ± 29.10), and in N:P (F_29,90_ = 12.74, P < 0.0001; range: 9.89 ± 2.27 to 43.27 ± 2.27).

### Univariate regressions with latitude

**Figure 1:**
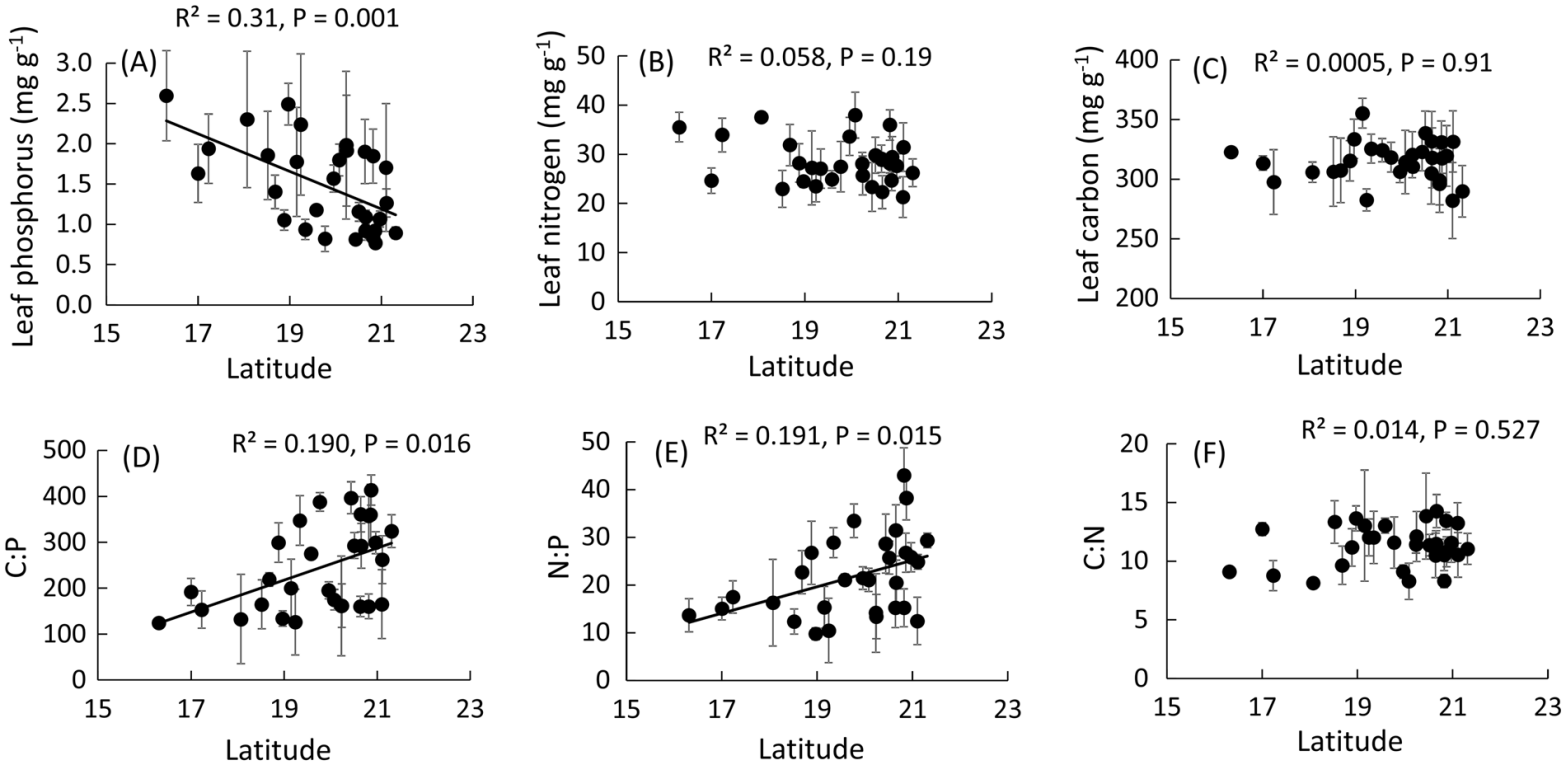
Relationship between latitude and (**A**) the concentration (mg g^−1^ d.w.) of leaf phosphorus, (**B**) leaf nitrogen, (**C**) leaf carbon, (**D**) carbon to phosphorus ratio (C:P), (**E**) nitrogen to phosphorus ratio (N:P), and (F) carbon to nitrogen ratio (C:N) for *Ruellia nudiflora* populations sampled along a 5° latitudinal transect from northern Yucatan (Mexico) to southern Belize (N = 30). R^2^ values, P-values and predicted relationships are from simple regressions in each case. Each dot represents a population mean ± standard deviation.

We found a significant negative relationship between the concentration of leaf P and latitude, where P concentrations increased towards lower latitudes (Fig. 1A). In contrast, we found no significant association between latitude and either leaf N or C (Fig. 1B,C). Results for element ratios indicated significant latitudinal gradients for C:P and N:P where both of these ratios decreased towards lower latitudes (Fig. 1D,E), but no association between C:N and latitude (Fig. 1F).

### Multivariate regressions assessing underlying abiotic factors

Results from multiple regression analyses indicated that precipitation (i.e. PC precipitation, see *Methods* section) was the only factor retained in the model for leaf P concentration (Table 1), describing a positive effect of this aspect of climate on this nutrient (i.e. higher precipitation was associated with higher leaf P). A subsequent multiple regression including both latitude and precipitation indicated a non-significant effect of latitude on leaf P (*t* = −1.85, P = 0.08), suggesting that latitudinal variation in this trait was underlain by precipitation. For leaf N, we found that soil cation exchange capacity (CEC) and clay content and soil C (i.e. PC1 soil and PC2 soil, respectively see *Methods*) were retained in the model but only the latter had a significant (negative) effect (Table 1), where high levels of soil C were associated with lower concentrations of leaf N. For leaf C, we found that temperature (i.e. PC temperature, see *Methods*), precipitation (PC precipitation), and soil C (PC2 soil) were retained in the model, but only the latter had a significant (positive) effect (Table 1) where increased soil C levels were associated with higher concentrations of C in leaves. Precipitation was marginally significantly (positively) associated with leaf C (Table 1).

**Table 1:** Results from multiple regressions testing for the effects of climatic factors (“PC temperature” and “PC precipitation”, *z*-scores from a Principal Components Analysis [PCA] of temperature- and precipitation-related variables, see *Methods*) and soil factors (“PC1 soil” and “PC2 soil”, *z*-scores from the first two PCs from a PCA using a set of soil variables, see *Methods*) on the concentration (mg g^−1^ d.w.) of phosphorus, nitrogen, carbon, and their ratios in leaves of *Ruellia nudiflora* sampled across 30 populations spanning 5° latitude (from SE Mexico and Belize).

Predictor	Phosphorus		Nitrogen		Carbon		C:P		N:P		C:N	
	Model *R*^2^ = 0.229		Model *R*^2^ = 0.267		Model *R*^2^ = 0.197		Model *R*^2^ = 0.144		Model *R*^2^ = 0.114		Model *R*^2^ = 0.262	
	*β*	*r* ^2^	*β*	*r* ^2^	*β*	*r* ^2^	*β*	*r* ^2^	*β*	*r* ^2^	*β*	*r* ^2^
PC temperature	—	—	—	—	−4.723	0.078	—	—	—	—	—	—
PC precipitation	0.262	0.229**	—	—	6.271	*0*.*110*	−27.53	*0*.*094*	—	—	—	—
PC1 soil	—	—	−0.853	0.045	—	—	—	—	2.734	*0*.*114*	—	—
PC2 soil	—	—	−2.199	0.240*	8.409	0.180*	—	—	—	—	0.963	0.262**

*R*^2^ = coefficient of determination, β = slope estimator, *r*^2^ = partial correlation coefficient (in the case of models including only one predictor this is equivalent to model *R*^2^). Significant (*P < 0.05, **P < 0.01) and marginal (0.05 < P < 0.10) effects are in bold and italics, respectively. Results are presented only for predictors retained after AIC model selection (see section on statistical analyses in the *Methods*).

For leaf element ratios, we found that precipitation was the only factor retained in the C:P model and exhibited a marginally significant negative effect on this ratio (Table 1). A subsequent multiple regression including both latitude and precipitation indicated a non-significant effect of latitude (*t* = 1.78, P = 0.09), suggesting that latitudinal variation in this ratio was accounted for to some extent by this aspect of climate. For the N:P ratio, the only factor retained in the model was soil CEC and clay content (i.e. PC1 soil) which had a marginally significant (positive) effect on this ratio (Table 1). A subsequent regression model including both latitude and this PC1 soil indicated that the effect of latitude was non-significant (*t* = 1.61, P = 0.12), suggesting that latitudinal variation in N:P was largely accounted by these soil features. Finally, soil C (PC2 soil) was the only factor retained in the C:N model and had a significant (positive) effect on this ratio (Table 1).

## Discussion

The studied *R*. *nudiflora* populations exhibited substantial variation in leaf C, N, P, and in their ratios. We found a significant cline of decreasing P with increasing latitude and no clines for N or C. With respect to elemental ratios, we found clines of increasing N:P and C:P with latitude, but no cline for C:N. Analysis of abiotic factors underlying these patterns indicated that leaf N was negatively associated with soil C content, whereas leaf C was positively associated with soil C. In addition, the latitudinal gradients in leaf P and C:P were largely accounted for by precipitation, the gradient in leaf C:P was mostly accounted by soil C, and the gradient in leaf N:P was accounted by soil CEC and clay content. These findings suggest that leaf element concentrations and their relative amounts are shaped by different abiotic factors, and that the simultaneous consideration of such factors is necessary to achieve a better understanding of how abiotic components of the environment shape plant stoichiometry.

Plant population variation in leaf N and P may reflect either genetic differentiation in functional or physiological traits or phenotypic plasticity in response to differences in biotic or abiotic conditions across sites. Previous research with *R*. *nudiflora* has shown that relatively weak evidence of local adaptation to abiotic factors such as soil conditions^24^, and genetic analyses indicate that this species has undergone a recent geographic expansion and that populations in the Yucatan Peninsula are of relatively recent origin^25^. These results suggests that there has not been enough time to foster strong genetic differentiation in plant functional traits and stoichiometry in this species. Considering that this species occurs in disturbed sites under a wide range of climatic and soil conditions, these results point at phenotypic plasticity as a predominant driver of population variation in leaf N and P for this species. Nonetheless, before reaching conclusions further studies involving common garden experiments under controlled conditions (e.g., Oleksyn *et al*.^17^), on-site manipulations (e.g., Lovelock *et al*.^9^), or reciprocal transplants across sites (e.g., Pennings *et al*.^26^) combined with genetic analyses (e.g., de Villemereuil *et al*.^27^) are necessary to quantify the relative contributions of genetic differentiation and phenotypic plasticity to leaf trait geographic variation in this species.

The observed increase in leaf P concentration with decreasing latitude found for *R*. *nudiflora* runs counter to previous large-scale studies across numerous plant taxa reporting decreases in plant P towards lower latitudes^6,7^. In addition, precipitation was the main factor accounting for the latitudinal gradient in leaf P concentration (positive relationship), whereas previous studies have reported that plant P latitudinal clines are associated primarily with temperature and, to a lesser extent, precipitation^6–8^. In this sense, infrequent and low precipitation limits soil weathering, organic matter production, and mineralization, leading to slower P release from soils^28^, which could explain the observed decrease in leaf P with decreasing precipitation toward higher latitudes. It also important to note that a much greater change in precipitation than in temperature along the sampled gradient likely conferred greater statistical power to detect effects of precipitation on leaf P (while potentially underestimating effects of temperature). On the other hand, our results indicated no evidence for a latitudinal gradient in leaf N for *R*. *nudiflora* as well as no association between this nutrient and climatic factors. These results were somewhat surprising, considering that although soils in the Yucatan Peninsula are considered N-limited, previous work in this region has found that soil N concentrations increase with precipitation^29^. Given that there is a sharp precipitation gradient along the studied transect, our results suggests other biotic or abiotic mechanisms are at work and counteracted the effects of precipitation on leaf N.

There was no evidence either for a latitudinal gradient in leaf C in *R*. *nudiflora*, and population variation in this trait was positively associated with soil C content. Although soil conditions exert a strong influence on leaf nutrient concentrations, particularly leaf N and P, the direction of causation may be different for leaf C, where plant C inputs via accumulation of biomass and decomposition may be a strong driver of organic soil C levels^30^. Thus, while feedbacks between soil and plant elements are prevalent in all cases, leaf N and P may be under stronger “bottom-up” control than C, and plant C may in turn contribute more strongly to soil C storage relative to plant N and P contributions to soil nutrient pools (albeit some exceptions as in the case of N_2_-fixing plants).

Patterns for element ratios indicated significant latitudinal gradients for N:P and C:P, which can be explained at first hand by the negative latitudinal cline in leaf P concentration combined with a lack of latitudinal gradients in leaf N and C. The observed decrease in N:P towards lower latitudes runs counter to previous work reporting a negative association between this ratio and latitude^6,7^. A closer examination of abiotic factors potentially driving these gradients indicated that precipitation partially explained the latitudinal gradient in C:P (values tended to decrease with increasing precipitation), possibly through controls of this aspect of climate on leaf P (see discussion above). In addition, N:P was not associated with climatic factors but was weakly positively associated with clayey soils with high pH. This might reflect the influence of increasing amounts of clay towards higher latitudes resulting in soils with smaller pore size, greater water retention, and therefore less N loss due to leaching and higher N uptake compared to plants growing in soils with lower clay content at lower latitudes. Although increasing precipitation towards lower latitudes could also lead to greater N loss through leaching, this interpretation is less likely given the lack of association between N and climatic factors as well as evidence form previous work reporting positive associations between N and precipitation for the study region^29^. It is also possible that the latitudinal gradient in leaf N:P was indirectly influenced by effects of precipitation on leaf P, but this influence of climate was not detectable after converting individual element data to ratios.

On the other hand, we found no evidence of latitudinal variation in C:N and population variation in this ratio was strongly positively associated only with soil C content, possibly due to the individual associations between soil C and leaf N and C (negative and positive, respectively). Therefore, contrary to leaf ratios including P (and presumably driven by latitudinal variation in this nutrient), spatial variation in leaf C:N appears to be uncoupled from latitudinal variation in abiotic conditions^28,31^, or in other words, the abiotic factors that govern population variation in this stoichiometric trait are not strongly associated with latitude. In combination, these results highlight that different aspects of leaf stoichiometry may vary in the strength or even presence of latitudinal effects and their underlying abiotic factors.

It is important to note that soil data used in the current work is at a spatial resolution of 1 by 1 km, which represents a relatively coarse scale to test for effects of spatial variation in soil features on population variation in leaf traits. In addition, the extrapolation methods used to obtain this soil database^32^ may yield different data relative to those obtained from on-site measurements, depending on the amount of sampling points available to perform extrapolations for a given region. Accordingly, data obtained from direct sampling at each site would have provided a more precise assessment of variation in soil characteristics and its association with population variation in leaf traits, particularly in the case of soil N and P concentrations which typically correlate strongly with leaf nutrients. Unfortunately, we did not collect soil samples at the study sites to obtain direct measurements of soil nutrients or physical properties. We do note, however, previous work from group which followed the same approach in a temperate system with an oak species^33^ also showed significant effects of soil variables on leaf traits at a similar spatial scale. These results suggest that there is broader-scale variation in soil conditions that is associated with population variation and such effects are being picked up by these analyses. Having said this, future work based on soil samples taken at the studied sites is necessary to gain a better understanding of soil effects on geographic variation in leaf nutrients and fully support the affirmations made based on the observed patterns.

Overall, results from this study call for increased attention to intra-specific clines in plant functional traits and nutrient stoichiometry. Identifying the factors and ecological mechanisms that govern intra-specific variation in plant nutrient stoichiometric is necessary to build a more robust understanding of how plant communities and ecosystems respond to environmental change as well as the contribution of organismal traits to ecosystem-level dynamics^22^. Determining the relative contributions of plasticity and genetic differentiation in stoichiometric traits along ecological gradients is also essential for addressing the potential for species adaptation to changing abiotic conditions.

## Methods

### Study species

*Ruellia nudiflora* (Acanthaceae) is a short-lived perennial herb (lifespan: 2-3 years) distributed from southern Texas (USA) to Honduras. It measures between 20 and 50 cm in height and grows in disturbed open areas or partially shaded sites on forest edges^24,34,35^. Historical accounts suggest that it has become naturalized in the tropical deciduous forests of the Yucatan Peninsula (Mexico), where it usually occurs at the edges of secondary forests or in human-made clearings^23^. It flowers from June to November, though the flowering peak is typically in July or August and is strongly correlated with the onset and amount of rainfall during the summer^36^. Fruits are dehiscent capsules and seeds disperse ballistically upon capsule explosion^33^. Previous work has shown that this species copes with water stress by shedding its leaves during drought periods and is tolerant to high temperatures^37^.

### Field sampling and measurements

In July 2013, we surveyed 30 populations of *R*. *nudiflora* distributed from northern Yucatan (Mexico) to southern Belize (see Table S1, *supplementary material*) spanning five degrees in latitude from 16°N to 21°N (ca. 900 km). The sampled transect spanned one-third of the latitudinal distribution range of *R*. *nudiflora*^35^ (14°N to 29°N) and covered the entire precipitation gradient and one-third of the temperature gradient experienced by this plant throughout its distribution range^23^. From north to south along this latitudinal gradient, there was more than four-fold increase in precipitation (700 to 2900 mm per year), a 20% decrease in coefficient of variation in precipitation (among months), and a decrease of 2 °C in mean annual temperature (from 26 to 24 °C) (climatic data obtained from: http://www.worldclim.org/). At each population, we selected four plants (N = 120 plants) and for each one we collected three fully expanded and undamaged leaves for quantification of leaf nutrients. For these same plants we also quantified structural (trichomes) and chemical (phenolic compounds) defences and results for these traits are reported elsewhere (see Abdala-Roberts *et al*.^20^). Although herbivory may be correlated with leaf nutrients^7,38^ and stoichiometry^39^, prior analyses indicated that leaf herbivory was not associated with any of the measured nutrients or their ratios (Pearson *r* = −0.001 to 0.125, P = 0.50 to 0.99). This allowed us to test for latitudinal variation in leaf nutrients and its underlying abiotic correlates without the confounding influence of herbivory. Leaves were placed in plastic bags, kept in a cooler with ice at 5 °C, and transported to the laboratory. All populations were sampled during a two-week period at the middle of the wet season to preclude phenological differences in leaf nutrient concentrations^20^.

### Quantification of leaf nutrients

To quantify leaf N and P, we digested approximately 0.3 g of grounded leaf material in a mixture of selenous sulphuric acid and hydrogen peroxide^40^. Diluted aliquots of the digestion were analysed by colourimetry for quantification of N (indophenol blue method) and phosphorus (molybdenum blue method) concentration using a Biorad 650 microplate reader (Bio-Rad Laboratories, Philadelphia, PA, USA) at 650 nm and 700 nm, respectively^40^. Leaf C content was determined by digestion of 0.2 g of dry leaf tissue in a mixture of potassium dichromate and sulphuric acid for 30 minutes in a block digester preheated to 155 °C. Diluted aliquots of the digestion were analysed by colorimetry using a microplate reader (Bio-Rad Laboratories) at 600 nm^41^. Glucose standards were included for calibration. For the statistical analyses, we used the concentration of each nutrient as mg g^−1^ tissue on a dry weight basis.

### Geographic, climatic and soil variables

We defined the geographic coordinates of each *R*. *nudiflora* population using a Global Positioning System device (Garmin, Kansas, USA). To characterize the climatic and soil conditions for each population, we used data from a subset of the bioclimatic variables in the WorldClim database (http://www.worldclim.org/) and data for soil variables from the SoilGrids database (http://www. soilgrids1km.isric.org), both at a 1 km grid resolution. Specifically, for climatic variables we used BIO1 (annual mean temperature, °C), BIO4 (temperature seasonality, expressed as the standard deviation of temperature among months*100), BIO5 (maximum temperature of the warmest month, °C), BIO6 (minimum temperature of the coldest month, °C), BIO12 (annual precipitation, mm), BIO13 (precipitation of the wettest month, mm), BIO14 (precipitation of the driest month, mm), BIO15 (precipitation seasonality, expressed as standard deviation of precipitation across months) as climatic variables^42^. For soil variables, we used depth to bedrock (R horizon) up to maximum 240 cm, bulk density (kg m^−3^), cation exchange capacity (cmolc kg^−1^), volume of coarse fragments (cm^−3^), organic carbon stock (tonnes per ha), organic carbon content (parts per thousand), pH, percentage of clay, percentage of silt and percentage of sand. Data for all the soil variables were obtained from the topsoil (2.5 cm depth). The procedures used to calculate these climatic and soil variables are fully described in Hijmans *et al*.^43^ and Hengl *et al.*^32^, respectively.

### Statistical analyses

We initially ran general linear models testing for population variation in N, P, C, C:P, C:N, and N:P using plant-level data (N = 120 plants). Then, to assess the presence of latitudinal variation, we performed population-level simple linear regressions (N = 30 populations) including latitude as predictor and using mean values for each variable by averaging across plants within each population. We also ran simple linear regressions between latitude and the within-population coefficient of variation (CV) for all leaf traits and in all but one case results were non-significant (R^2^ ≤ 0.081, P ≥ 0.14). The only exception was the C:P ratio, for which the within-population CV increased towards higher latitudes (R^2^ = 0.25, P = 0.01). This suggests that latitudinal patterns in intra-specific variation did not introduce a bias in our population-level analyses of latitudinal gradients in the leaf traits studied, and also that the population-level analyses did not mask patterns of intra-population variation.

To investigate the influence of abiotic factors on latitudinal variation in leaf nutrients and their ratios, we performed population-level multiple regressions (N = 30) including climatic (temperature- and precipitation-related) and soil variables as predictors. In each of these cases, predictors were dropped or retained based upon model selection using AIC values^44^. To make use of the information from all climatic and soil variables without inflating Type I error due to multiple tests, we previously summarized climatic and soil variables using Principal Components Analysis (PCA) and used the *z*-scores from the PCs in the multiple regressions. We summarized climatic variables by conducting two PCAs, one for temperature variables and one for precipitation variables. In each case, climatic variables were summarized with the first principal component. The first principal component explained 79% of the variance in the four temperature variables across populations (PC temperature) and was positively related to annual mean annual temperature, temperature seasonality, and maximum temperature of the warmest month, and negatively related to minimum temperature of the coldest month. Similarly, the first principal component explained 77% of the variance in the four precipitation variables across populations (PC precipitation), and was positively related to annual precipitation, precipitation of the wettest month, and precipitation of the driest month. We used the standardized *z*-scores of the first principal component (PC hereafter) from each ordination in the above multiple regressions testing for the effect of abiotic factors on leaf traits. Latitude was significantly negatively associated with PC precipitation (Pearson *r* = 0.78, *P* < 0.001) but not to PC temperature (*r* = 0.20, *P* = 0.32), whereas PC temperature and PC precipitation were unrelated (*r* = 0.26, *P* = 0.17). Likewise, for soil variables we performed a PCA where the first two axes explained 76% of the variance in the 10 selected soil variables across populations. The first principal component for soil variables (PC1 soil) was positively related to cation exchange capacity (CEC), pH and percentage of clay, whereas the second principal component for soil variables (PC2 soil) was positively related to organic C stock and content. As for the climate-related PCs, we used the *z*-scores of these soil PCs in the above multiple regressions. Latitude was significantly positively associated with PC1 soil (*r* = 0.80, P < 0.0001), but was not associated with PC2 soil (*r* = 0.27, P = 0.15). In addition, PC temperature and PC precipitation were negatively associated with PC1 soil (*r* = −0.36, P = 0.04 and *r* = −0.72, P < 0.0001, respectively), and PC precipitation (but not PC temperature) was negatively associated with PC2 soil (*r* = −0.49, P = 0.005).

Finally, we assessed which abiotic factors underlie latitudinal variation in each of the leaf  traits by running multiple regressions including latitude and factors that (a) exhibited a significant latitudinal gradient (i.e., PC precipitation, PC1 soil, see above) and (b) were retained after stepwise removal in the above multiple regressions testing for the influence of abiotic factors on a given leaf trait. We ran these models only for leaf traits that exhibited a significant latitudinal gradient based upon the simple linear regressions with latitude. If the effect of latitude (based on the simple regressions) was non-significant after accounting for the selected abiotic factor(s), we interpreted this as evidence that such factor(s) underlie(s) the latitudinal gradient for that trait.

Throughout the results section, we present means and S.E. as descriptive statistics. All regressions were conducted with PROC REG, general linear models in PROC GLM, and the PCAs using PROC FACTOR (rotation = varimax) in SAS 9.4^45^. Normality of residuals was previously verified and met in all cases. Stepwise selection of predictors in the multiple regressions was conducted using the FORWARD option in PROC REG.

## Electronic supplementary material

Supplementary information
