# Supplementary material for: Intra-Specific Latitudinal Clines in Leaf Carbon, Nitrogen, and Phosphorus and their Underlying Abiotic Correlates in Ruellia Nudiflora
Source: Sci Rep. 2018 Jan 12;8:596. doi: 10.1038/s41598-017-18875-w (PMC5766631; doi:10.1038/s41598-017-18875-w)
Supplement: Supplementary file 1 — Supplementary information [file 41598_2017_18875_MOESM1_ESM.pdf]

INTRA-SPECIFIC LATITUDINAL CLINES IN LEAF CARBON, NITROGEN, AND PHOSPHORUS AND THEIR UNDERLYING ABIOTIC CORRELATES IN *Ruellia nudiflora*

Running head: Latitudinal gradients in leaf nutrients

Luis Abdala-Roberts[1*], Felisa Covelo[2], Víctor Parra-Tabla[1], Jorge C. Berny Mier y Terán[3], Kailen A. Mooney[4], and Xoaquín Moreira[5*]

[1]Departamento de Ecología Tropical, Campus de Ciencias Biológicas y Agropecuarias, Universidad Autónoma de Yucatán, Apartado Postal 4-116, Itzimná, 97000 Mérida, Yucatán, México.

[2] Departamento de Sistemas Físicos, Químicos y Naturales, Universidad Pablo de Olavide, Carretera de Utrera km. 1, 41013 Sevilla, Spain.

[3]Department of Plant Sciences, University of California-Davis, One Shields Avenue, Davis, California, USA 95616

[4]Department of Ecology and Evolutionary Biology, University of California, Irvine, California, USA 92697.

[5]Misión Biológica de Galicia (MBG-CSIC), Apdo. 28, 36080 Pontevedra, Spain.

*Corresponding author:
Email: abdala.luis@yahoo.com or xmoreira1@gmail.com

Phone/fax Number: +52 999 9423206

Supplementary material

Table S1. Geographical coordinates (decimal degrees) and climatic characteristics (mean annual temperature in °C and mean annual precipitation in mm) for 30 populations of *Ruellia nudiflora* sampled from northern Yucatan (Mexico) to southern Belize. Y = Yucatan (Mexico); Q = Quintana Roo (Mexico); B = Belize. Climatic data are from World Clim (Hijmans et al. 2005).

Population	Latitude (N)	Longitude (W)	Prec. (mm)	Temp (°C)
San Crisanto (Y)	21.311667	89.1774	676	25.7
Mocochá (Y)	21.106389	89.4403	929	25.7
Cosgaya (Y)	21.096944	89.7046	725	25.6
Tetiz (Y)	20.966668	89.9229	748	25.9
San Pedro Chimay (Y)	20.869722	89.534	984	25.9
Celestun (Y)	20.863611	90.1364	816	26.3
San José Tzal (Y)	20.821389	89.6568	1024	25.9
Xtepen (Y)	20.818611	89.7353	1004	26.0
Uayalceh (Y)	20.660833	89.6026	1021	26.1
Punta Laguna (Q)	20.645556	87.6318	1216	25.4
Telchaquillo (Y)	20.635556	89.4602	1060	26.0
Sacalum (Y)	20.505	89.5929	997	26.1
Lolche (Q)	20.435278	87.6569	1179	25.6
Loltun (Y)	20.240556	89.4596	1105	25.7
Tixmeuac (Y)	20.228611	89.1231	1083	25.8
Tzucacab (Y)	20.080278	89.0651	1064	25.9
Catmis (Y)	19.956389	88.9494	1074	25.9
María Morelos (Q)	19.766111	88.7223	1251	26
Chunhunub (Q)	19.58167	88.5921	1173	25.8
Presidente Juarez (Q)	19.339722	88.5505	1128	25.8
Nuevo Israel (Q)	19.239444	88.5501	1115	25.8
La Pantera (Q)	19.146944	88.4929	1147	25.7
Lazaro Cardenas (Q)	18.967778	88.2122	1431	25.8
Buenavista (Q)	18.88	88.2381	1445	25.8
Bacalar (Q)	18.677	88.4012	1100	25.7
Huay pix (Q)	18.516667	88.4249	1247	25.5
Orange Walk (B)	18.066667	88.5614	1540	25.1
Belmopan (B)	17.23	88.7809	2025	24.8
Hope Creek (B)	17.0025	88.315	2263	25.2
Indian Creek (B)	16.31222	88.834	2904	25.1
